# Not your usual neurodegenerative disease: a case report of neuronal intranuclear inclusion disease with unconventional imaging patterns

**DOI:** 10.3389/fnins.2023.1247403

**Published:** 2023-08-10

**Authors:** Luyao Xu, Hongxia Zhang, Hanye Yuan, Liwen Xie, Junliang Zhang, Zhigang Liang

**Affiliations:** Department of Neurology, Yantai Yuhuangding Hospital Affiliated to Qingdao University, Yantai, China

**Keywords:** neuronal intranuclear inclusion disease, Susac syndrome, skin biopsy, genetic testing, diffusion weighted imaging, characteristic imaging features

## Abstract

**Background:**

Neuronal intranuclear inclusion disease (NIID) is a rare neurodegenerative illness with characteristic brain magnetic resonance imaging (MRI) manifestations: diffuse symmetric white-matter hyperintensities in lateral cerebral ventricle areas in fluid-attenuated inversion recovery (FLAIR) and high-intensity signals along the corticomedullary junction of the frontal–parietal–temporal lobes in diffusion weighted imaging (DWI). Here, we report a case of adult-onset NIID who was misdiagnosed with Susac syndrome (SS) due to unusual corpus callosum imaging findings.

**Case presentation:**

A 39-year-old man presented with chronic headache, blurred vision, tinnitus, and numbness in the hands as initial symptoms, accompanied by cognitive slowing and decreased memory. Brain MRI revealed round hypointense lesions on T1-weighted imaging (T1WI) and hyperintense lesions on T2WI/FLAIR/DWI in the genu and splenium of the corpus callosum. An initial diagnosis of SS was made based on the presence of the SS-typical symptoms and SS-characteristic radiology changes. Furthermore, the patient’s symptoms improved upon completion of a combined pharmacotherapy plan. However, no significant changes were evident 18 months after the brain MRI scan. Eventually, the patient was then diagnosed with NIID based on a skin biopsy and detection of expanded GGC (guanine, guanine, cytosine) repeats in the NOTCH2NLC gene.

**Conclusion:**

The present NIID case in which there was simultaneous onset of altered nervous and visual system functioning and atypical imaging findings, the atypical imaging findings may reflect an initial change of NIID leukoencephalopathy.

## Introduction

Neuronal intranuclear inclusion disease (NIID) is a slowly progressive neurodegenerative condition that is characterized by eosinophilic hyaline intranuclear inclusions in the central and peripheral nervous system, as well as in the visceral organs (Takahashi-Fujigasaki, 2003; Sone et al., 2016). The etiology and pathogenesis of NIID have not been clarified. Immunohistochemically, intranuclear inclusions are positive for ubiquitin and ubiquitin related proteins, including p62, SUMO1, FUS, MYO6, and OPTN-C, suggesting the ubiquitin-proteasome pathway in the nucleus plays an important role in NIID (Kimber et al., 1998; Pountney et al., 2003; Mori et al., 2011, 2012; Nakamura et al., 2014). Recently, the GGC repeat expansions in the 5′UTR of the NOTCH2NLC gene have been identified as the pathogenic mutation of adult- and juvenile-onset NIID (Fiddes et al., 2018; Suzuki et al., 2018; Deng et al., 2019; Sone et al., 2019).

The onset of the disease varies from infancy to approximately between the ages of 60–70 years (Takahashi-Fujigasaki et al., 2016) and can be classified into three categories: infantile, juvenile, and adult NIID (Takumida et al., 2017). However, current knowledge of the clinical features of adult-onset NIID is lacking. The clinical characteristics of adult-onset NIID have been reported as follows: pyramidal and extrapyramidal symptoms, cerebellar ataxia, dementia, convulsions, neuropathy, sensory disturbance, and autonomic dysfunction (Sone et al., 2016; Liu et al., 2019). It is difficult to make a conclusive early diagnosis of NIID due to the disease’s highly variable clinical symptoms, signs, and onset age. As a result, NIID is often misdiagnosed. In recent years, brain changes indicated by MRI, combined with skin biopsy pathological results and genetic testing [fragile X mental retardation 1 (FMR1) or Notch 2 N-terminal-like C (NOTCH2NLC) testing] can contribute to the accurate diagnosis of NIID.

Here, we report an adult-onset case of NIID in which the patient’s nervous and visual systems were affected simultaneously at onset and the patient had atypical imaging findings of lesions in the corpus callosum. Susac syndrome (SS) was diagnosed initially due to symptom characteristics at disease onset resembling the SS-typical symptom triad and the observation of SS-characteristic radiology changes. An absence of radiology changes for 18 months after the diagnosis of SS had been made led to further investigation, which yielded a corrected diagnosis of NIID based on the identification of a NIID-associated NOTCH2NLC gene variant and the findings of skin biopsy.

## Case description

The case included in the current study was that of a 39-year-old man with no family history of NIID or any other neurodegenerative disease. He was admitted to the hospital in May 2020 after primarily complaining of headache (sharp pain), paroxysmal blurred vision (no abnormalities were found in visual acuity or color vision), tinnitus, and numbness of the hands over the preceding 6 months. Upon admission to the hospital, cognitive slowing and impaired memory function were detected by neurological examination; no abnormalities were found in other physical examinations. The following relevant scales were completed: the Mini-Mental State Examination scale (score, 27/30), the Montreal Cognitive Assessment scale (score, 27/30), and Barthel Index measure of activities of daily living (score, 100).

The results of blood biochemistry, routine blood panel, anti-endothelial cell antibody, anticardiolipin antibody, anti-cyclic citrullinated peptide antibody, antikeratin antibody, antiperinuclear factor, and routine and biochemical cerebrospinal fluid tests were all normal.

**Figure 1:**
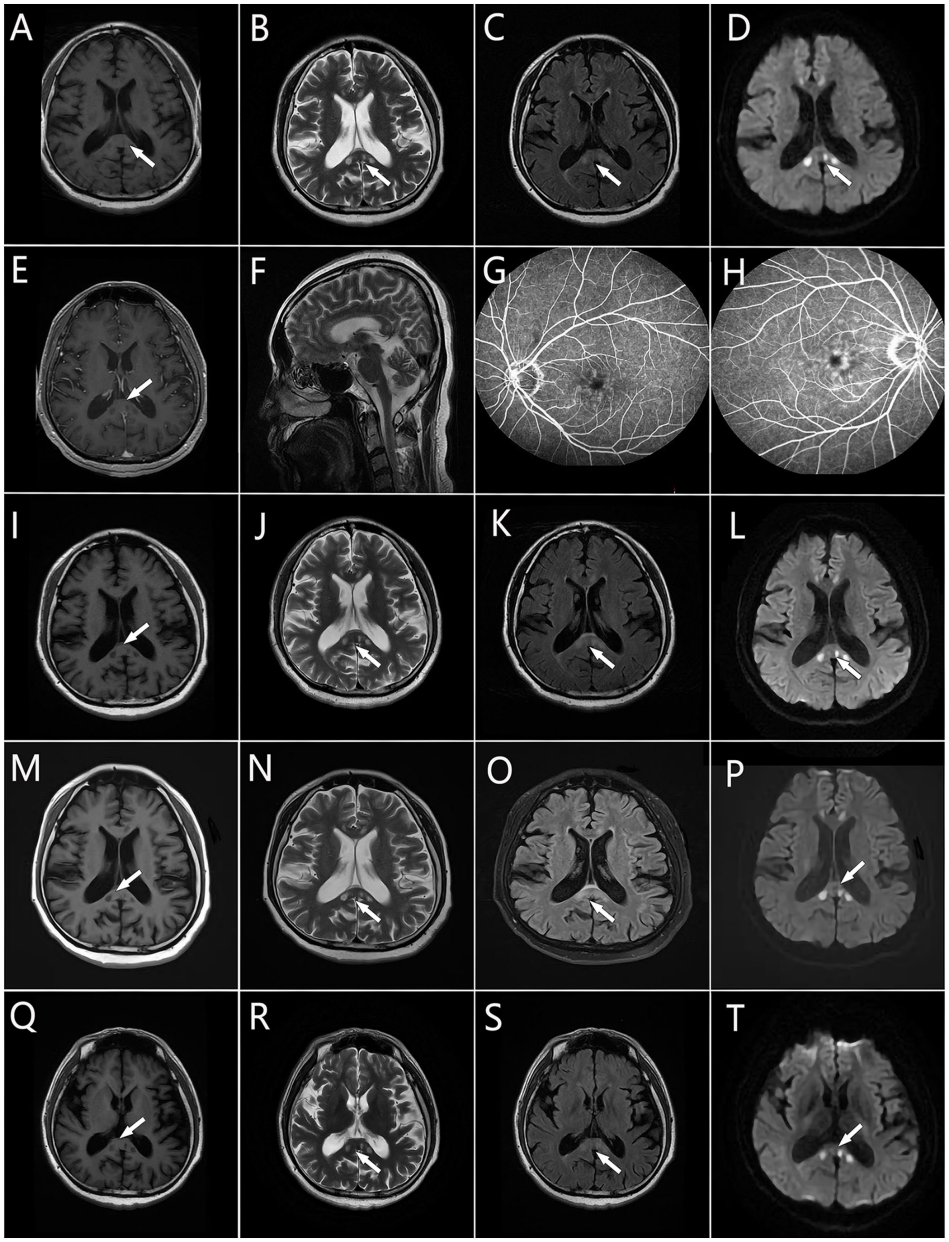
Brain MRI. **(A,I,M,Q)** Multiple round hypointense lesions on T1 in the corpus callosum. **(B,F,J,N,R)** Multiple round hyperintense lesions on T2 in the corpus callosum. **(C,K,O,S)** Multiple round hyperintense lesions on FLAIR located in the corpus callosum. **(D,L,P,T)** Multiple hyperintense lesions on DWI located in the corpus callosum. **(E)** Multiple hyperintense lesions on contrast-enhanced MR located in the corpus callosum. **(G,H)** The macular capillary was dilated and there is fluorescein leaking from the dilated blood vessels. Areas of hyperintensity or hypointensity are denoted by the white arrows.

An MRI scan of the head revealed multiple round hypointense lesions on T1-weighted imaging (T1WI) (Figure 1A) and hyperintense lesions on T2WI/fluid-attenuated inversion recovery (FLAIR) analysis (Figures 1B,C,F) in the genu and splenium of the corpus callosum. On diffusion-weighted imaging (DWI) (Figure 1D), multiple round hyperintense lesions were found to be located in the genu and splenium of the corpus callosum. Contrast-enhanced MRI revealed multiple round hypointense lesions on T1WI and hyperintense lesions on T2WI in the genu and splenium of the corpus callosum; no lesion enhancements were found (Figure 1E). The patient’s electroencephalogram demonstrated paroxysmal short-range (5–7 Hz) slow waves in the eye-closed resting state. Fundus fluorescein angiography (FFA) of the (left and right) eyes (Figures 1G,H) demonstrated the presence of a dilated macular capillary and fluorescein leakage from dilated blood vessels. The patient had not experienced any hearing loss and there were no abnormal otolaryngological findings.

Anamnesis suggested that the patient’s nervous, visual, and auditory systems were likely to have been affected simultaneously, an onset of disease symptoms similar to that seen with SS. Moreover, the symptoms themselves resembled the typical SS symptom triad (encephalopathy, visual disturbances, and hearing loss) (Lim et al., 2004), and SS-characteristic radiology changes (Egan, 2019) were observed. Notably, multiple round hyperintense lesions in the corpus callosum were evident on DWI, a finding that is an important indicator for SS diagnosis (Egan, 2019). The patient’s neurological symptoms improved upon completion of a combined pharmacotherapy plan consisting of high-dose intravenous methylprednisolone (80 mg/d for 5 d), oral aspirin (an anti-platelet agent, 100 mg/d for 14 d), and oral idebenone tablets (a synthetic analogue of the antioxidant ubiquinone used for neuroprotection and cognitive enhancement, 90 mg/d for 3 months). Subsequently, the patient was given a maintenance prescription of oral methylprednisolone (1 mg/kg bodyweight with slow tapering) and idebenone tablets (90 mg/d). After 2 weeks, the patient’s headache and blurred vision symptoms gradually improved, and he was discharged from the hospital.

One month after being discharged, a brain MRI showed multiple round hypointense lesions on T1WI (Figure 1I) and hyperintense lesions on T2WI/FLAIR/DWI (Figures 1J–L) in the genu and splenium of the corpus callosum; no significant changes were observed in the lesions, and the patient had fully recovered, except for a slightly impaired memory. Treatment was continued with oral methylprednisolone, and the dosage was gradually decreased. Three months after the patient’s discharge from the hospital, his brain MRI findings remained unchanged relative to the diagnostic MRI findings (Figures 1M–P), but the patient had no disease symptoms or signs at this time.

The patient was readmitted to the hospital in April 2021 after complaining of a paroxysmal visual field defect, headache (sharp pain), and hypoesthesia of the right hand. Cognitive slowing and reduced tendon reflex responses were found by neurological examination; no abnormalities were observed during other neurological examinations. The findings of his brain MRI (Figures 1Q–T) and contrast-enhanced MRI remained unchanged from his initial admittance. His electroencephalogram showed paroxysmal short-range (4–7 Hz) slow waves in the eyes-closed resting state; following hyperventilation, slow waves with a slower rhythm and sharp slow waves (epileptic waves) appeared. His nerve conduction velocity revealed no peripheral nerve damage, but F-wave latencies were prolonged in both lower limbs. Electromyography showed no signs of neurogenic injury or of myogenic lesion.

**Figure 2:**
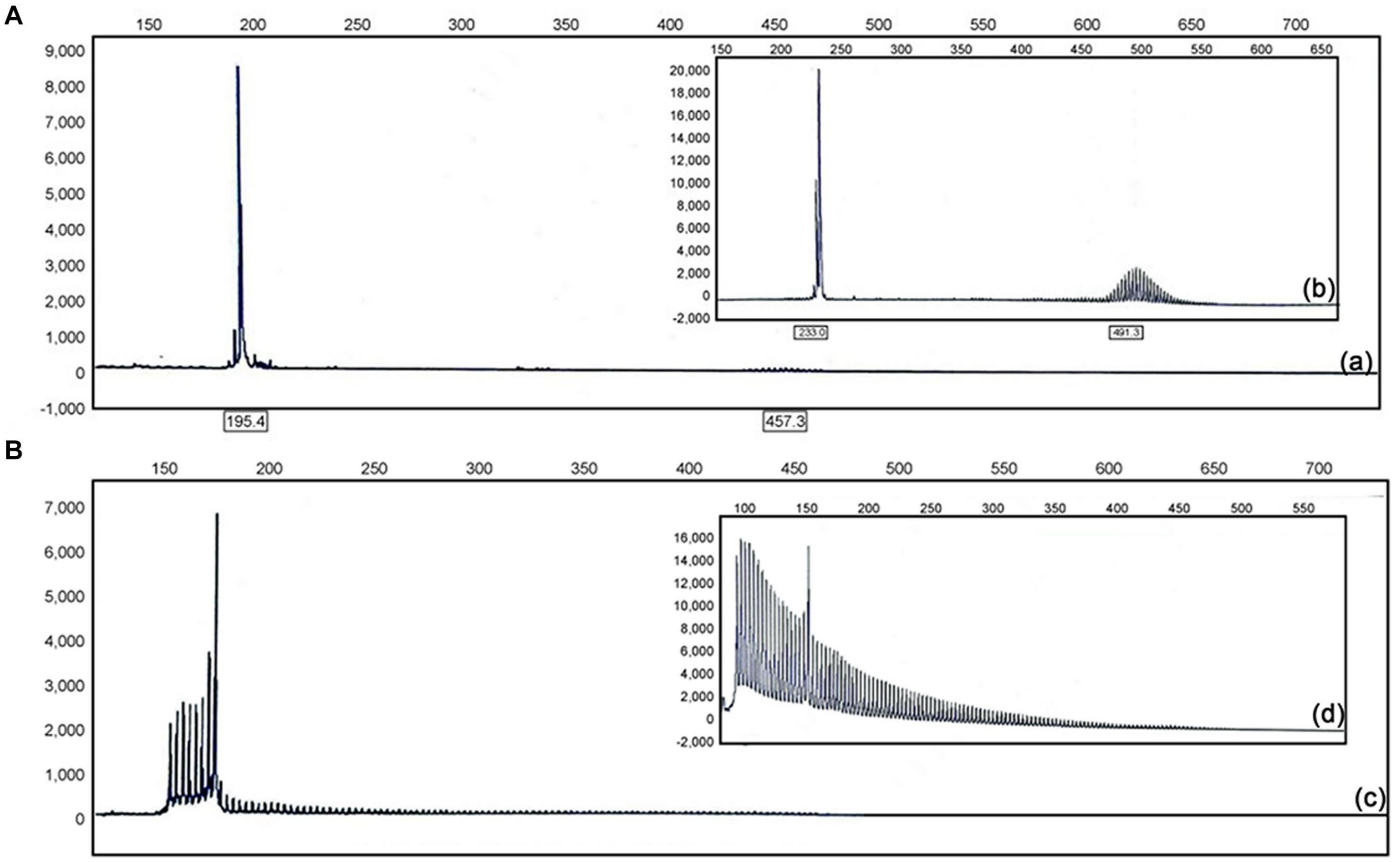
**(A)** Electropherograms showing GC-rich regions using PCR and repeat-primed PCR assays in affected individuals (a) and positive control subjects (b). **(B)** GC-rich PCR showed the patient had 101 GGC repeats in the 5′ UTR of NOTCH2NLC (c); GC-rich PCR showed the GGC repeats in the 5′ UTR of NOTCH2NLC in positive control subjects (d).

Genetic testing was conducted with repeat-primed polymerase chain reaction (RP-PCR) and guanine–cytosine-rich PCR (GC-PCR). We observed a 101 GGC (guanine, guanine, cytosine) repeat in the 5′-untranslated (5’UTR) region of the NOTCH2NLC gene [normal range < 40 (Tian et al., 2019)] [Figures 2B(c)]. A skin biopsy was performed via light microscopy (3DHistech) on samples obtained above the patient’s ankle and thigh; hematoxylin and eosin staining revealed eosinophilic spherical inclusion bodies within some sweat gland epithelial cell nuclei (Supplementary Figure S1). However, the patient refused to undergo immunohistochemical staining (p62 and ubiquitin staining) due to reasons of its own. Based on these findings, the patient was given a revised diagnosis of NIID. He was then prescribed symptomatic treatment, including oral analgesics (sibelium, 10 mg/d for 14 days) and oral idebenone tablets (90 mg/d for 3 months). Over the subsequent months, the patient reported a gradual relief of his symptoms. Follow-up by telephone 18 months after the patient was discharged revealed that the patient no longer had a paroxysmal visual field defect or hypoesthesia of the hands and that he suffered from only occasional headaches.

**Figure 3:**
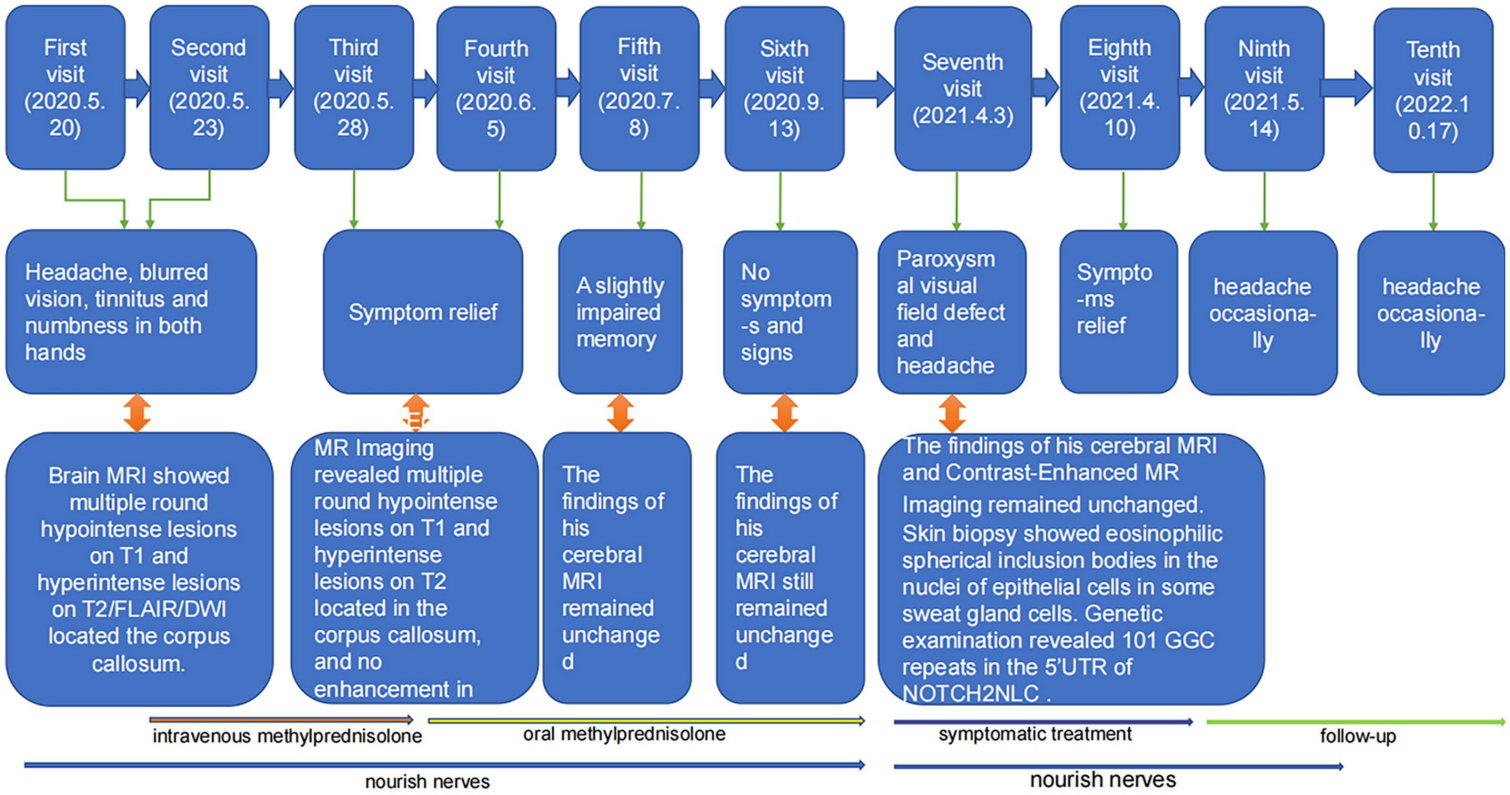
The timeline of the patient’s course of disease.

The timeline of the patient’s disease course is shown in Figure 3.

## Discussion

NIID is a clinically heterogeneous illness with a range of clinical manifestations. For adult-onset NIID, dementia was the most prominent initial symptom, followed by ataxia and unconsciousness (Sone et al., 2016). Affected patients may present with central nervous system impairments, including dementia (Munoz-Garcia and Ludwin, 1986; Araki et al., 2016), cerebellar ataxia (Patel et al., 1985), epilepsy (Shindo et al., 2019), paroxysmal disturbances in consciousness (Zhang et al., 2022), Parkinsonism (Liu et al., 2008; López-Blanco et al., 2019) and abnormal mental behavior (Chi et al., 2020). Adult NIID may also present with peripheral nerve damage (Sone et al., 2005), including limb weakness and sensory disturbances. Other symptoms of autonomic nervous system involvement may include bladder dysfunction, miosis, syncope, and vomiting (Sone et al., 2005; Nakamura et al., 2018). According to published case reports, some patients have presented with subacute progressive encephalitis (Huang et al., 2021), stroke-like symptoms (Lin et al., 2020), mental abnormalities (Chi et al., 2020), and dopa-responsive dystonia (Paviour et al., 2005). The present report presents a case of adult-onset NIID in which the patient presented with the symptoms of headache, blurred vision, and tinnitus as initial symptoms, accompanied by numbness in the hands, cognitive slowing, and impaired memory.

Previously, brain MRI in patients with NIID has revealed diffuse symmetric white-matter hyperintensities in lateral cerebral ventricle areas in FLAIR images (Sone et al., 2014; Yu et al., 2019) and high-intensity signals along the corticomedullary junction of the frontal–parietal–temporal lobes in DWI (Sone et al., 2016; Yu et al., 2019). These findings, which are considered to be characteristic imaging manifestations of NIID, are known as the cortical linear sign and the subcortical lace sign, respectively. It has been suggested that the high-intensity DWI signal may be continuous across the corticomedullary junction in NIID patients, with lesions being confined mostly to the frontal lobe in early-stage NIID (Abe and Fujita, 2017). With progression of the disease, the high-intensity DWI signal may develop gradually without extending into the deep white matter (Nakamura et al., 2018; Omoto et al., 2018). However, high-intensity signal in the corticomedullary junction has been reported to disappear a few years after being observed (Kawarabayashi et al., 2018). Thus DWI findings may be dynamic across NIID phases. As such, it is vital that follow-up procedures be conducted in patients whose MRI scans indicate changes. The presently described patient’s brain MRI revealed multiple small round hypointense lesions on T1WI and hyperintense lesions on T2WI/FLAIR/DWI located in the genu and splenium of the corpus callosum, and no significant changes were found after 18 months of follow-up. Some patients with NIID have shown high-intensity signal in the splenium of the corpus callosum only during an early disease stage (Yu et al., 2019; Wang et al., 2020b; Li et al., 2022), with subsequent gradual development of signal in the region of the corticomedullary junction as the disease progressed (Wang et al., 2020a). Therefore, we speculate that the atypical imaging manifestations of our patient may be attributed to his young age at which diffuse white matter lesions have not yet become evident. If so, then the small asymmetric round lesions observed could reflect an initial change of NIID leukoencephalopathy. What’s more, compared with the patients with typical MRI findings, the presently described patient’s symptoms are mild. Therefore, the atypical imaging findings have the potential to guide early diagnosis, so as to take early intervention to delay the progression of NIID.

Skin biopsy is feasible and helpful for the diagnosis of NIID. The pathological hallmarks are eosinophilic hyaline intranuclear inclusions in neuronal and non-neuronal central nervous system cells, in autonomic nervous system peripheral cells, as well as in visceral organs and skin (Sone et al., 2011, 2014). Intranuclear inclusions are round substances with a perinuclear diameter of 1.5–10 μm. They are ubiquitin- and p62-positive, consisting of fibrous materials lacking membranous structures when viewed under electron microscopy (Bitto et al., 2014; Sone et al., 2014). Currently, the identification of the GGC repeat expansion at the 5′ end of NOTCH2NLC as the genetic cause of NIID will help in identifying the molecular pathogenesis in NIID. In NIID, the GGC repeat ranges from 66 to over 500; healthy people have fewer than 43 repeats. To date, there is no association between the GGC repeat size and the severity or the age of onset of the disease (Ishiura et al., 2019; Sone et al., 2019; Tian et al., 2019). In this case, genetic testing revealing a NOTCH2NLC allele with a GGC repeat expansion in the 5’UTR region, and the concomitant observation of eosinophilic inclusion bodies in sweat gland cells, both of them affirmed a diagnosis of NIID together.

Furthermore, in this case, FFA of the eyes showed dilated macular capillary and fluorescein leakage from dilated blood vessels. Dilation of macular capillaries leads to an expansion of intercellular space and increased vessel permeability, thereby enabling fluorescein leakage from dilated capillaries. This phenomenon may lead to retinal abnormalities over time. Retinal abnormalities, including dilated macular capillary, may be a very early retinal change in NIID (Liu et al., 2022; Sone et al., 2023).

There is, in addition, one further point to make. For NIID with corpus callosum lesions, it is mainly differentiated from Reversible splenial lesion syndrome (RESLES) and Marchiafava Bignami disease (MBD). In this case, the patient was initially misdiagnosed as SS due to the presence of the SS-typical symptoms and SS-characteristic radiology change. However, in the subsequent progression of SS, branch retinal artery occlusions and sensorineural hearing loss can also be seen, which is not available in NIID, which will help to distinguish the two.

There is not yet a standard treatment for NIID. Symptomatic treatment may improve a patient’s quality of life and delay the progress of some symptoms. Analgesics have been reported to be typically effective in relieving the occurrence of NIID headache/migraine (Wang et al., 2020a). Steroid hormones have been used in the treatment of paroxysmal encephalopathy, but there is no clear evidence indicating that the drug can improve prognosis (Sone et al., 2016). In some cases, the treatment of intravenous methylprednisolone may improve the symptoms of dementia in patients with acute disease onset (Yadav et al., 2019). In the treatment of NIID with encephalitis-like symptoms, dehydration therapy can reduce brain edema and relieve symptoms. For infantile NIID or juvenile NIID with extrapyramidal disorder, there have been reported cases of dopamine treatment, but there is no clear efficacy (Paviour et al., 2005; Lai et al., 2010; Yoshimoto et al., 2017).

## Conclusion

This paper presents a NIID case in which the nervous and visual systems were affected simultaneously in early phase of the disease. The brain MRI showed multiple round hypointense lesions in T1WI analysis and hyperintense lesions in T2WI/FLAIR/DWI analysis in the corpus callosum. And the FFA of the eyes showed dilated macular capillary and fluorescein leakage from dilated blood vessels. Referring to previous literature, we consider the small asymmetric round lesions observed could reflect an initial change of NIID leukoencephalopathy, and the dilated macular capillary may be a very early retinal change in NIID. All in all, this report on a case of NIID may be conducive to enriching clinicians’ understanding of the disease based on its particular clinical manifestations and imaging characteristics.

## Data availability statement

The original contributions presented in the study are included in the article/Supplementary material, further inquiries can be directed to the corresponding author.

## Ethics statement

The studies involving humans were approved by the Ethics Committee of Yuhuangding Hospital. The studies were conducted in accordance with the local legislation and institutional requirements. The participants provided their written informed consent to participate in this study. Written informed consent was obtained from the individual(s) for the publication of any potentially identifiable images or data included in this article.

## Author contributions

LyX drafted and revised the manuscript. HZ collected patient information and revised the manuscript. ZL collected and analyzed patient information. HY, LwX, and JZ interpreted the data and edited the manuscript. All authors contributed to the article and approved the submitted version.

## Funding

This work was partially supported by grants from the Yantai Science and Technology Plan Project (2021YD033 and 2018SFGY092).

## Conflict of interest

The authors declare that the research was conducted in the absence of any commercial or financial relationships that could be construed as a potential conflict of interest.

## Publisher’s note

All claims expressed in this article are solely those of the authors and do not necessarily represent those of their affiliated organizations, or those of the publisher, the editors and the reviewers. Any product that may be evaluated in this article, or claim that may be made by its manufacturer, is not guaranteed or endorsed by the publisher.
