# Supplementary figures and images for: Not your usual neurodegenerative disease: a case report of neuronal intranuclear inclusion disease with unconventional imaging patterns
Source: Front Neurosci. 2023 Aug 10;17:1247403. doi: 10.3389/fnins.2023.1247403 (PMC10447982; doi:10.3389/fnins.2023.1247403)

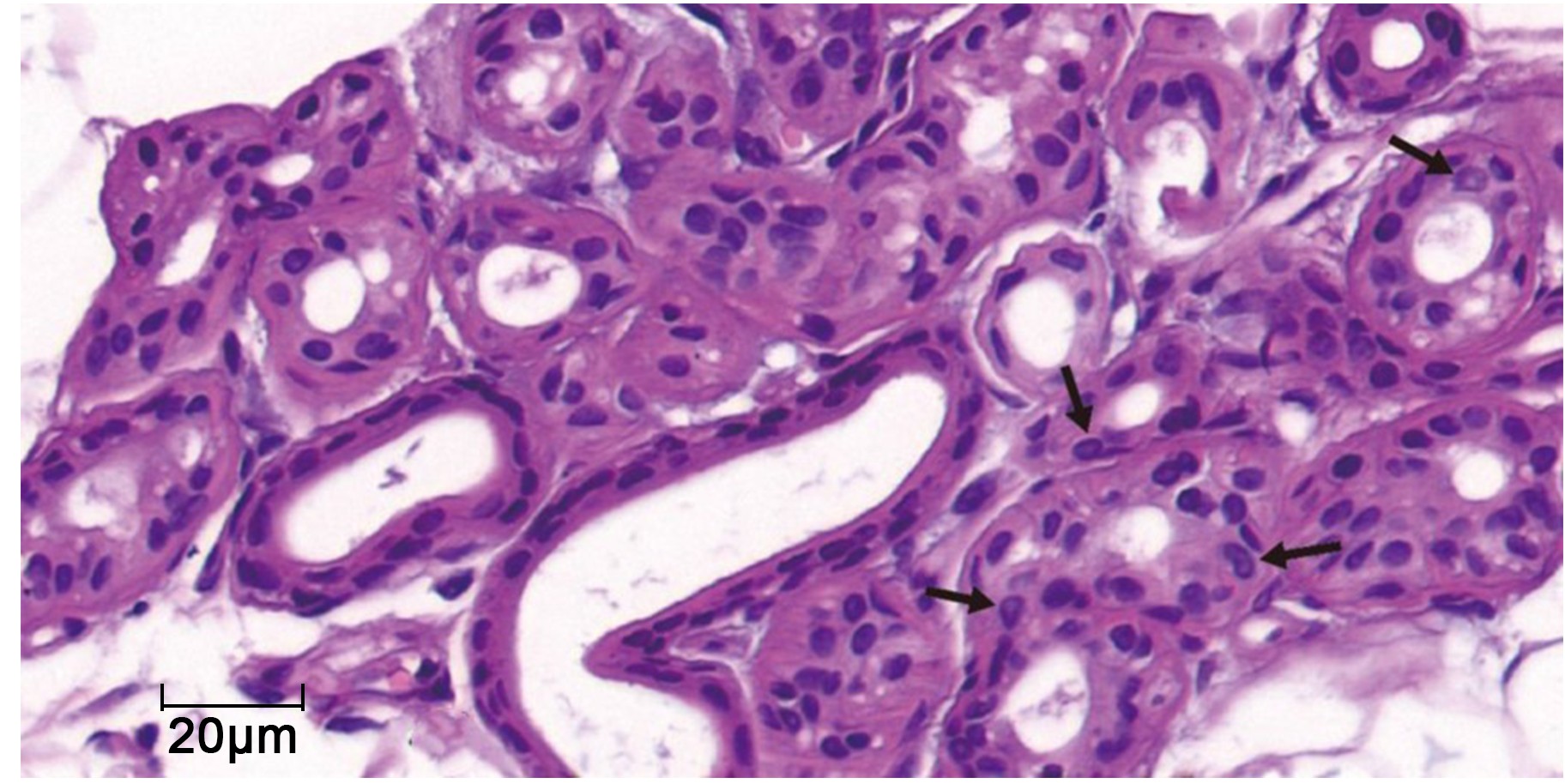

Supplement: Supplementary file 1 [file Image_1.jpeg]

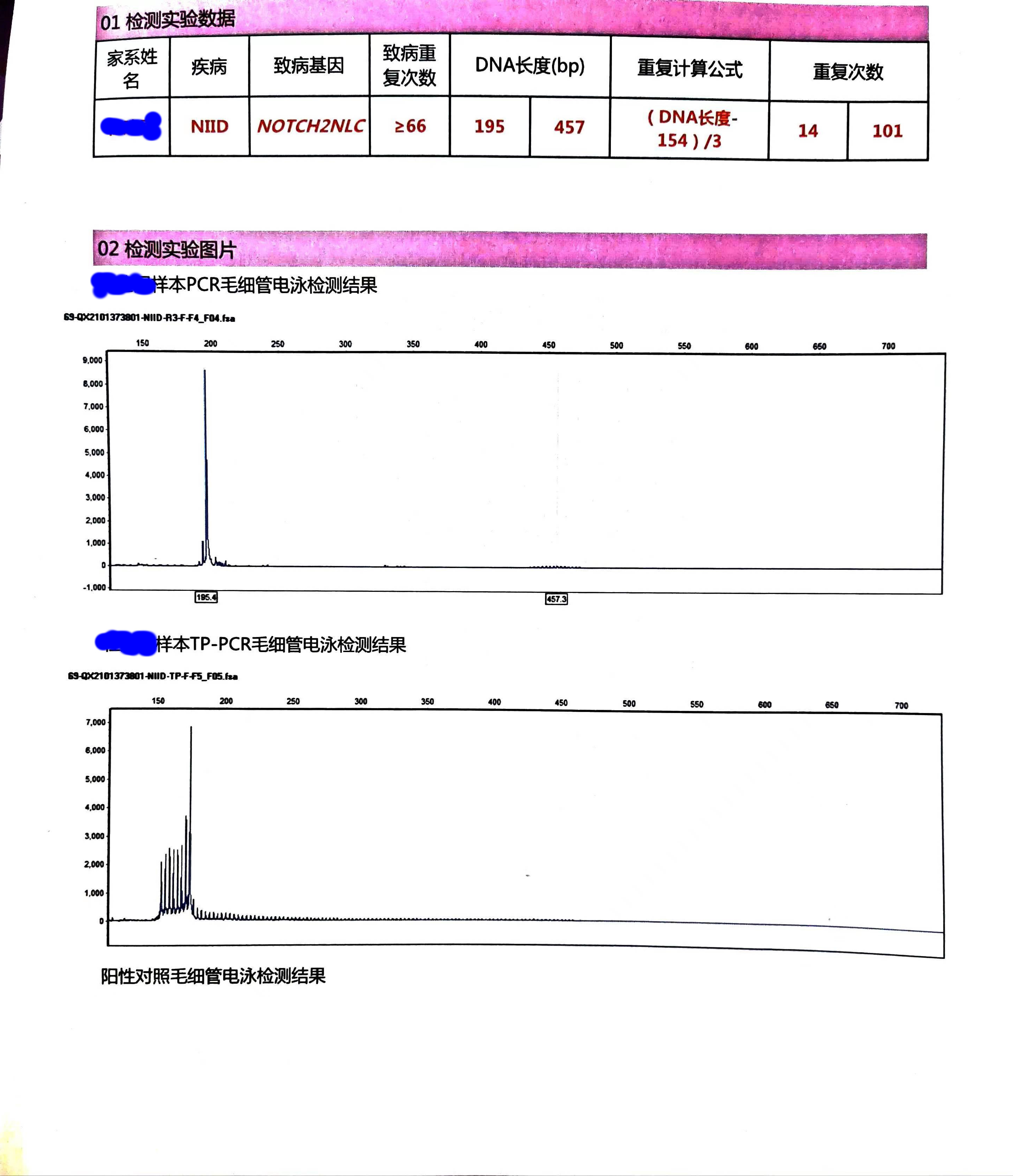

Supplement: Supplementary file 2 [file Image_2.jpeg]

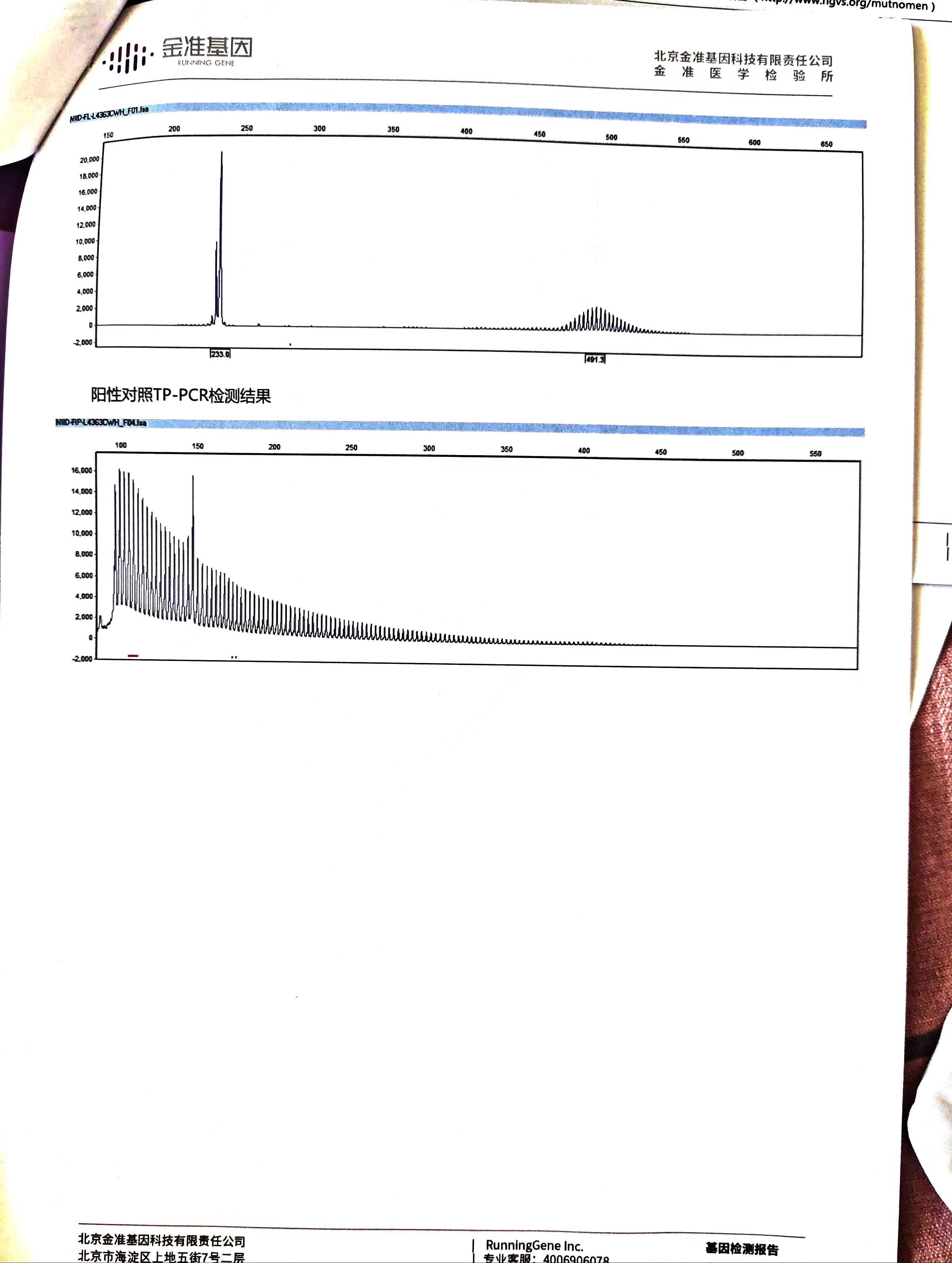

Supplement: Supplementary file 3 [file Image_3.jpeg]
